# Fungus Hæmatodes

**Published:** 1845-02

**Authors:** 


*Fungus hæmatodes.*—At a meeting of the Sheffield Medical Society, the president exhibited a preparation of fungus hæmatodes, arising in the antrum, and passing into the mouth. Its growth was very rapid; and the hæmorrhage had been very frequent, and to a great extent, so as to produce very great exhaustion. It was determined to tie the carotid as a preliminary step to the removal of the fungus, but the system was so prostrated that the removal of the diseased growth was not attempted. The patient died three hours after the artery was tied. The antrum was completely filled by the fungus, which passed outwards, and then turned inwards, filling the palate, and resting on the tongue, impeding deglutition and speech. The floor of the orbit showed the commencement of absorption, and the sella turcica also presented the appearance of disease.—*Ibid.*

